# Acceleration of 2D-MR fingerprinting by reducing the number of echoes with increased in-plane resolution: a volunteer study

**DOI:** 10.1007/s10334-020-00842-8

**Published:** 2020-04-04

**Authors:** Yusuke Yokota, Tomohisa Okada, Yasutaka Fushimi, Akira Yamamoto, Satoshi Nakajima, Koji Fujimoto, Sonoko Oshima, Gregor Koerzdoerfer, Mathias Nittka, Josef Pfeuffer, Kaori Togashi

**Affiliations:** 1 Department of Diagnostic Imaging and Nuclear Medicine, Kyoto University, 54 Shogoin Kawaharacho, Sakyoku, Kyoto, 606-8507 Japan; 2 Human Brain Research Center, Graduate School of Medicine, Kyoto University, 54 Shogoin Kawaharacho, Sakyoku, Kyoto, 606-8507 Japan; 3 Magnetic Resonance, Siemens Healthcare GmbH, Henkestrasse 127 Postfach 32 60, 91050 Erlangen, Germany

**Keywords:** Magnetic resonance imaging, Brain, Fingerprinting, Test–retest reliability

## Abstract

**Objective:**

To compare the absolute values and repeatability of magnetic resonance fingerprinting (MRF) with 3000 and 1500 echoes/slice acquired in 41 s and 20 s (MRF3k and MRF1.5k, respectively).

**Materials and methods:**

MRF3k and MRF1.5k scans based on fast imaging with steady precession (FISP) were conducted using a 3 T scanner. Inter-scan agreement and intra-scan repeatability were investigated in 41 and 28 subjects, respectively. Region-of-interest (ROI) analysis was conducted on T1 values of MRF3k by two raters, and their agreement was evaluated using intraclass correlation coefficients (ICCs). Between MRF3k and MRF1.5k, differences in T1 and T2 values and inter-measurement correlation coefficients (CCs) were investigated. Intra-measurement repeatability was evaluated using coefficients of variation (CVs). A *p* value < 0.05 was considered statistically significant.

**Results:**

The ICCs of ROI measurements were 0.77–0.96. Differences were observed between the two MRF scans, but the CCs of the overall ROIs were 0.99 and 0.97 for the T1 and T2 values, respectively. The mean and median CVs of repeatability were equal to or less than 1.58% and 3.13% in each of the ROIs for T1 and T2, respectively; there were some significant differences between MRF3k and MRF1.5k, but they were small, measuring less than 1%.

**Discussion:**

Both MRF3k and MRF1.5k had high repeatability, and a strong to very strong correlation was observed, with a trend toward slightly higher values in MRF1.5k.

**Electronic supplementary material:**

The online version of this article (10.1007/s10334-020-00842-8) contains supplementary material, which is available to authorized users.

## Introduction

Magnetic resonance fingerprinting (MRF) is a recently introduced quantitative MRI framework that enables simultaneous measurement of multiple quantitative tissue parameters, such as T1 and T2 relaxation times. In MRF, acquisition parameters are varied in a pseudorandom manner so that different tissues have their own unique signal evolutions, which are compared with precalculated signal evolutions listed in a predefined dictionary to find the best match [1]. MRF has been used in brain disorders including brain tumors [2] and epilepsy [3, 4] and is expected to enable better comparisons among different sites and scanners [5–7].

In MRF, a large number of measurements has been considered necessary to robustly generate parameter maps. One MRF scan of a single slice using the established method acquires 3000 echoes and takes 42 s [8], resulting in a 14-min scan for a whole-brain acquisition covering 20 slices; however, a faster and higher-resolution acquisition is favorable for clinical application. Current MRF measurements of T1 and T2 have been reported to be very stable [9, 10], and an MRF scan with a higher resolution in a shorter scan time is considered feasible.

Based on the results of a phantom study, we hypothesized that a 1500-echo MRF scan with some additional increase in spatial resolution would yield reliable measurements comparable to those of a 3000-echo MRF scan (i.e., twice the number of echoes). Therefore, the repeatability of the two MRF scans was compared, and agreement between them was investigated in a relatively large number of subjects.

## Materials and methods

### Subjects

This study was approved by the institutional review board. Forty-three healthy subjects (23 men, mean age 23.6 years, range 20–29 years) were enrolled in this study, and written informed consent was obtained before scanning. The exclusion criteria were poor image quality, which was typically due to excessive motion during scanning; apparent slice shifts; large susceptibility artifacts; and any abnormal findings in the brain. Two subjects were excluded due to large susceptibility artifacts caused by metal dental work affecting the values in the brain parenchyma, and MRF images of 41 subjects were used for further analysis.

### MR measurements

A 3-T MR scanner (MAGNETOM Skyra, Siemens Healthcare, Erlangen, Germany) was used with a 32-channel head coil. Two-dimensional axial T2-weighted images parallel to the AC-PC plane were acquired with the following parameters: repetition time (TR), 5380 ms, echo time (TE), 99 ms; flip angle (FA), 150°, field of view, 230 mm × 230 mm; matrix size, 640 × 640; slice thickness, 5 mm with no inter-slice gaps. Based on the T2-weighted images, three slices showing the centrum semiovale, basal ganglia, and middle cerebellar peduncle (MCP) were selected, and MRF slices were placed at those slice positions. MRF scans were conducted using a prototype spiral fast imaging with steady precession (FISP) sequence [8] that has low sensitivity to magnetic field inhomogeneities. The trajectories were corrected using a one-time calibration and a generalized eddy-current model by Tan and Meyer [11]. Spiral trajectories were rotated by 82.5° between successive repetitions [12].

**Fig. 1:**
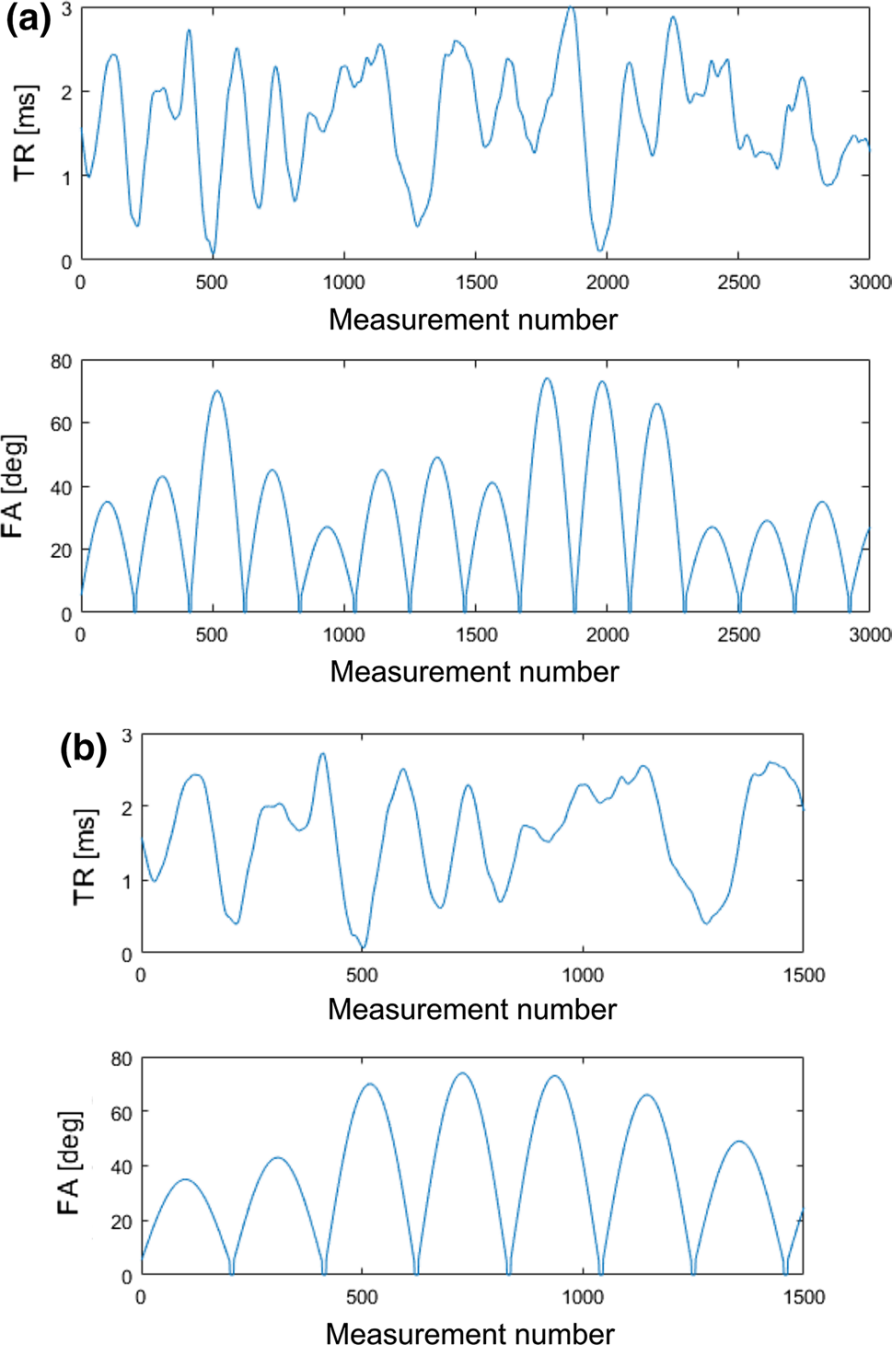
TRs and FAs for **a** MRF3k and **b** MRF1.5k. Changes in TR are the same, but different FA patterns were used for the two measurements

The slices were measured in a sequential, non-interleaved manner. Each slice acquisition consisted of an adiabatic, non-selective inversion pulse and a train of FISP echoes. Acquisition started at 21 ms after the inversion pulse with a TE of 2 ms and a base TR of 12 ms. TR and FA were continuously changed, ranging from 12.1 to 15.0 ms and from 0° to 74°, respectively (Fig. 1). MRF with 3000 echoes (MRF3k) [8, 10] and MRF with 1500 echoes (MRF1.5k) were conducted. The latter scan parameter was set based on a study using an NIST/ISMRM system phantom (https://www.nist.gov/programs-projects/quantitative-mri). Details are presented in the supplementary material.

**Fig. 2:**
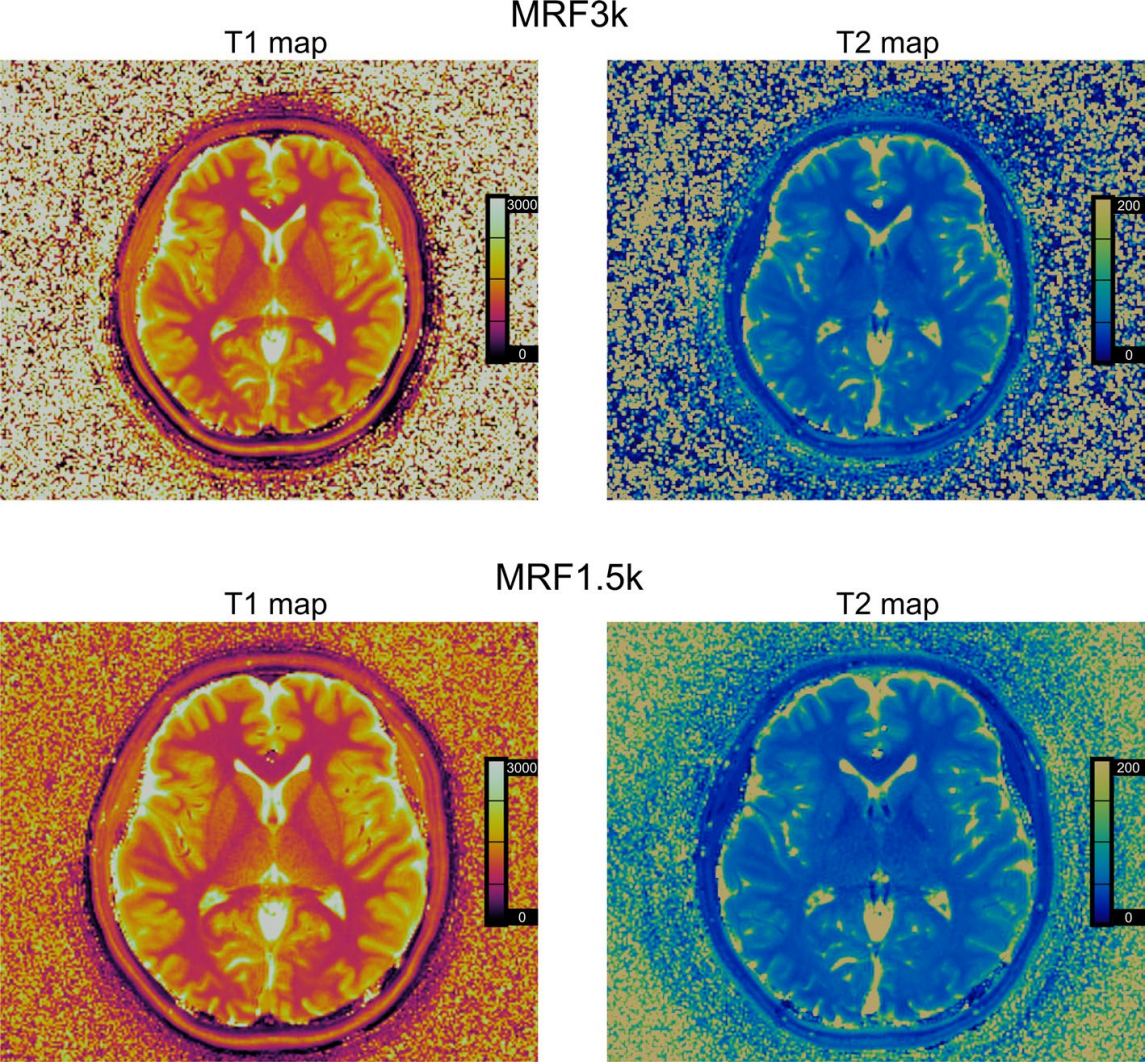
Representative MRF images of a subject

The scan time of each slice was 41 s and 20 s for MRF3k and MRF1.5k, respectively. For MRF3k, the field of view was 300 mm × 300 mm, and the in-plane resolution was 1.17 mm × 1.17 mm, for MRF1.5k, the values were 256 mm × 256 mm and 1.0 mm × 1.0 mm, respectively. The slice thickness was 5 mm for both scans. Before the MRF scans, an RF-field map [13] of the whole volume was acquired in 20 s and used during the reconstruction of both MRF scans. Each MRF scan was repeated twice in 28 out of the 41 subjects. Representative images are presented in Fig. 2.

The MRF dictionary was calculated for a range of discrete T1 and T2 values using the Bloch equation. The step sizes (ranges: min–max) were 10 ms (10–100 ms), 20 ms (100–1000 ms), 40 ms (1000–2000 ms) and 100 ms (2000–4500 ms) for T1 and 2 ms (2–100 ms), 5 ms (100–150 ms), 10 ms (150–300 ms), 50 ms (300–800 ms), 100 ms (800–1600 ms) and 200 ms (1600–3000 ms) for T2, which were the same for both MRF3k and MRF1.5k.

### Image pre-processsing and ROI analyses

After brain extraction, MRF1.5k T1 and T2 maps were registered to the MRF3k T1 map of the same slice for each subject in 2D mode (in-plane translation and rotation) and resampled to 1.17 mm × 1.17 mm in-plane resolution using FMRIB’s Linear Image Registration Tool (https://fsl.fmrib.ox.ac.uk/fsl/fslwiki).

**Fig. 3:**
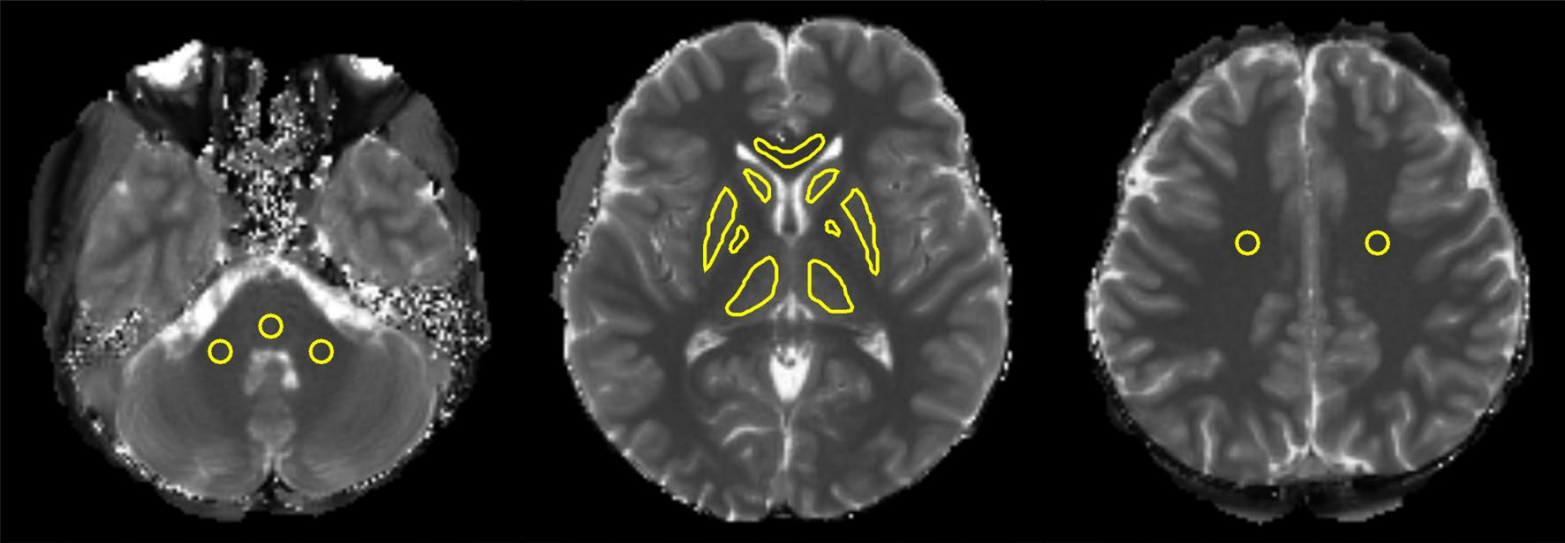
Multiple ROIs placed for analysis. ROIs were located in **a** the MCP, pons, **b** caudate head, putamen, globus pallidus, thalamus, corpus callosum, and **c** bilateral centrum semiovale

The following structures were selected for region-of-interest (ROI) analysis: centrum semiovale, caudate head, putamen, globus pallidus, thalamus, corpus callosum, middle cerebellar peduncle (MCP), and ventral part of the pons (Fig. 3). ROIs on the standard MRF3k T1 map images were drawn independently by two raters (rater 1: Y.Y. with 11 years and rater 2: S.N. with 15 years of experience as neuroradiologists) using ImageJ software (https://imagej.nih.gov/ij/). For some of the structures with apparent boundaries, i.e., the basal ganglia, caudate head, and thalamus, ROIs were drawn by tracing their inner boundaries. For the rest of the structures with no apparent boundaries, circular ROIs were placed. The mean T1 and T2 values of the ROIs were measured.

### Statistical analyses

The Shapiro–Wilk test was used to check the normality of the data distribution.

Inter-rater agreement in ROI placement was evaluated using the intraclass correlation coefficient (ICC) of the mean T1 values of two raters’ ROIs positioned on the MRF3k T1 maps. The ICC value is interpreted as a reliability scale in the following manner: less than 0.5, poor; 0.5–0.75, moderate; 0.75–0.9, good; greater than 0.90, excellent [14].

The inter-measurement differences in the mean T1 and T2 values of each of the ROIs were examined between MRF3k and MRF1.5k using paired *t* tests or Wilcoxon tests. Inter-measurement correlation analysis was also conducted using Pearson’s correlation coefficient or Spearman’s rank correlation coefficient. In the cases with repeated measurements, the mean of two measurement values was used. Biases of T1 and T2 values between MRF3k and MRF1.5k were assessed using Bland–Altman plots.

Intra-measurement repeatability was evaluated using coefficients of variation (CVs) of mean T1 and T2 values of repeated measurements for both MRF3k and MRF1.5k using the abovementioned ROIs. These CVs were compared between MRF3k and MRF1.5k using paired *t* test or Wilcoxon test.

Statistical significance was defined as *p* < 0.05. All statistics were calculated using MedCalc Statistical Software version 18 (MedCalc Software bvba, Ostend, Belgium).

## Results

### Inter-rater agreement in ROI placement

The ICCs of the T1 values in the ROIs of the two raters were 0.77–0.96. High agreement between them was confirmed (Table 1), and the ROIs of rater 1 were used thereafter.

**Table 1:** Intra-class correlation coefficient (ICC) of T1 values derived from 2 raters’ ROIs

ROIs	ICC
Left MCP	0.85
Right MCP	0.91
Pons	0.86
Left caudate	0.88
Right caudate	0.87
Left putamen	0.96
Right putamen	0.94
Left pallidus	0.81
Right pallidus	0.79
Left thalamus	0.84
Right thalamus	0.89
Corpus callosum	0.84
Left semiovale	0.86
Right semiovale	0.77

*MCP* middle cerebellar peduncle, *ROIs* regions-of-interest

### Inter-measurement difference in mean T1 and T2 values and their correlation

The mean or median T1 values of MRF3k and MRF1.5k were 870–919 ms and 862–910 ms, respectively, in the white matter (corpus callosum and centrum semiovale) and 1024–1381 ms and 1038–1397 ms, respectively, in the gray matter (caudate, basal ganglia and thalamus). The T2 values in MRF3k and MRF1.5k were 32.7–38.3 ms and 34.0–41.0 ms, respectively, at the cerebral white matter ROIs and 29.1–51.7 ms and 31.1–53.9 ms, respectively, in the deep gray matter ROIs. Some ROI values showed significant inter-measurement differences (Table 2).

**Table 2:** T1 and T2 values in regions-of-interest (ROIs) of rater 1

ROIs	MRF3k		MRF1.5k		*p* value
	T1 value	95% CI	T1 value	95% CI	
Left MCP	1022	1012–1032	1032	1020–1044	0.003*
Right MCP	1044	1034–1054	1045	1034–1055	0.875
Pons	1100^†^	1084–1111^†^	1123^†^	1113–1138^†^	< 0.001^†^*
Left caudate	1381	1369–1394	1397	1385–1409	< 0.001*
Right caudate	1349	1337–1362	1373	1359–1386	< 0.001*
Left putamen	1244	1233–1256	1263	1252–1274	< 0.001*
Right putamen	1226	1214–1237	1256	1245–1267	< 0.001*
Left pallidus	1053^†^	1032–1067^†^	1038^†^	1015–1054^†^	0.002^†^*
Right pallidus	1024	1013–1036	1039	1028–1051	< 0.001*
Left thalamus	1167	1148–1186	1168	1148–1188	0.595
Right thalamus	1150	1133–1168	1171	1152–1190	< 0.001*
Corpus callosum	870	861–879	862	852–872	0.006*
Left semiovale	919^†^	907–925^†^	910^†^	897–923^†^	0.001^†^*
Right semiovale	903^†^	889–918^†^	904^†^	889–918^†^	0.059^†^

ROIs	MRF3k		MRF1.5k		*p* value
	T2 value	95% CI	T2 value	95% CI	
Left MCP	39.9^†^	38.4–41.9^†^	43.7^†^	42.2–45.4^†^	< 0.001^†^*
Right MCP	40.2^†^	39.4–41.1^†^	43.3	42.5–44.2^†^	< 0.001^†^*
Pons	38.1^†^	36.2–40.0^†^	41.4^†^	40.0–43.3^†^	< 0.001^†^*
Left caudate	50.9	50.1–51.7	52.8	52.0–53.5	< 0.001*
Right caudate	51.7	50.9–52.4	53.9	53.1–54.6	< 0.001*
Left putamen	45.1^†^	44.2–45.7^†^	47.9^†^	47.3–48.5^†^	< 0.001^†^*
Right putamen	45.4	44.6–46.3	48.8	48.0–49.7	< 0.001*
Left pallidus	29.6^†^	29.1–30.2^†^	31.1^†^	30.4–31.5^†^	< 0.001^†^*
Right pallidus	29.1	28.4–29.7	31.4	30.8–32.1	< 0.001*
Left thalamus	41.8	41.1–42.4	44.3	43.5–45.0	< 0.001*
Right thalamus	41.4^†^	40.5–42.0^†^	44.4^†^	43.8–45.4^†^	< 0.001^†^*
Corpus callosum	32.7	32.0–33.5	34.0	33.3–34.7	< 0.001*
Left semiovale	38.1^†^	37.2–39.1^†^	41.0^†^	40.0–41.8^†^	< 0.001^†^*
Right semiovale	38.3^†^	37.2–39.2^†^	40.1^†^	39.7–40.9^†^	< 0.001^†^*

*CI *confidence interval, *MCP *middle cerebellar peduncle

*Statistical significance

^†^Non-normal distribution, and median values are presented, and non-parametric Wilcoxon test was used for comparison

**Fig. 4:**
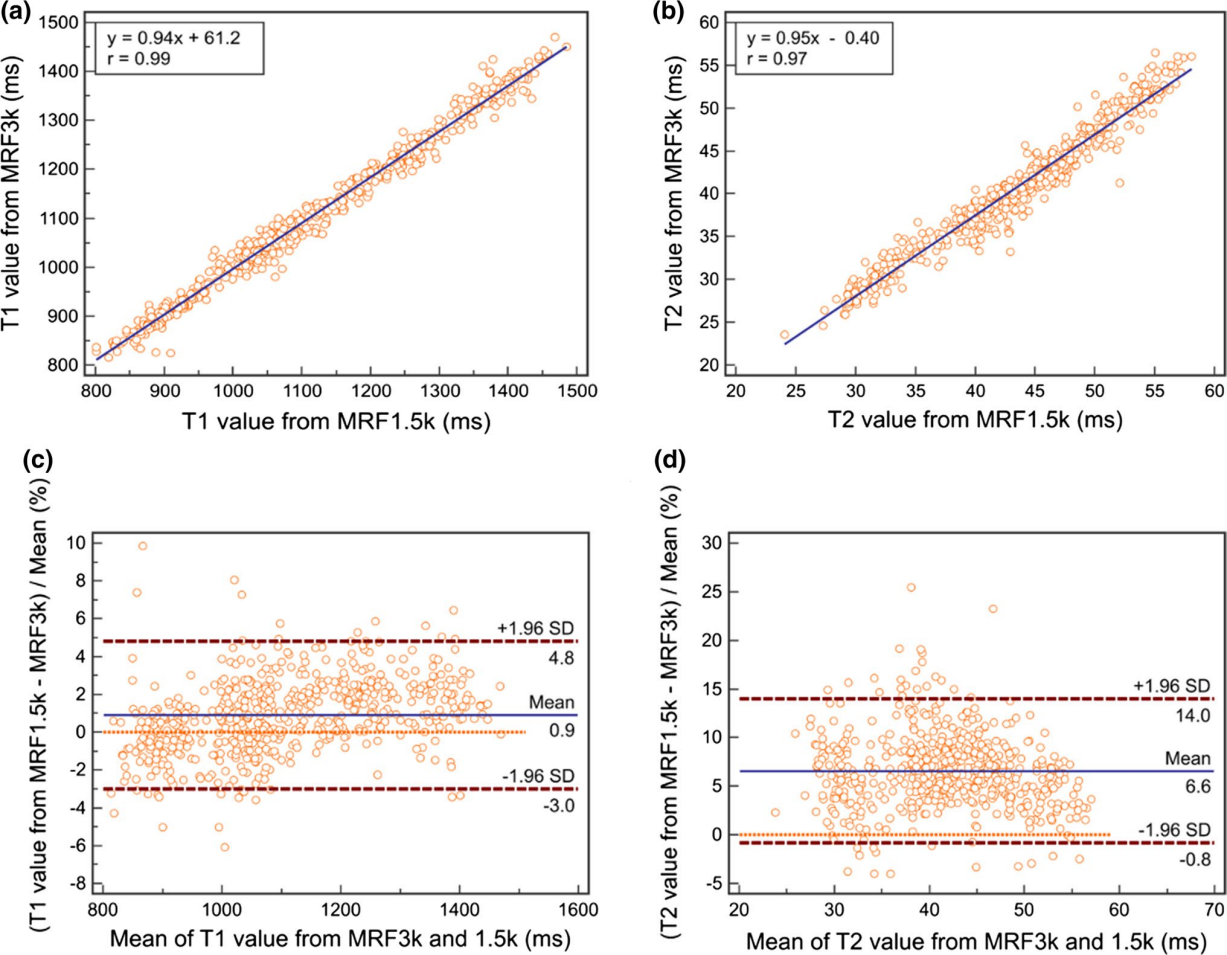
Correlation plots (top, **a** and **b**) and Bland–Altman plots (bottom, **c** and **d**) of overall ROI values between MRF3k and MRF1.5k for (right) T1 and (left) T2 values

Between MRF3k and MRF1.5k, the correlation coefficients of the measured T1 and T2 values of each ROI were 0.79–0.98 and 0.73–0.93, respectively, showing a strong to very strong correlation [15], and those of the overall ROI values were 0.99 and 0.97 for the T1 and T2 values, respectively (Table 3). Bland–Altman plots of T1 and T2 values showed relatively small differences in the mean percent differences (95% CI) of 0.9 (− 3.0 to 4.8) and 6.6 (− 0.8 to 14.0) for T1 and T2 values, respectively, between MRF3k and MRF1.5k (Fig. 4).

**Table 3:** Inter-measurement correlation coefficient (*r*) and 95% confidence interval (CI) between MRF3k and MRF1.5k

ROIs	T1 value		T2 value	
	*r*	95% CI	*r*	95% CI
Left MCP	0.88	0.78–0.93	0.83	0.71–0.91
Right MCP	0.84	0.71–0.91	0.85^†^	0.73–0.91
Pons	0.81	0.66–0.89	0.92	0.85–0.96
Left caudate	0.83	0.69–0.90	0.88	0.78–0.93
Right caudate	0.79	0.64–0.88	0.73	0.54–0.84
Left putamen	0.88	0.78–0.93	0.90	0.82–0.95
Right putamen	0.92	0.86–0.96	0.93	0.87–0.96
Left pallidus	0.80	0.65–0.89	0.88^†^	0.79–0.94
Right pallidus	0.88	0.79–0.94	0.90	0.82–0.95
Left thalamus	0.98	0.97–0.99	0.88	0.78–0.93
Right thalamus	0.97	0.95–0.99	0.89	0.80–0.94
Corpus callosum	0.86	0.74–0.92	0.83	0.71–0.91
Left semiovale	0.80	0.66–0.89	0.77	0.60–0.87
Right semiovale	0.87	0.76–0.93	0.79	0.63–0.88
Overall ROIs	0.99^†^	0.99–0.99	0.97^†^	0.97–0.98

*MCP *middle cerebellar peduncle, *ROIs *regions-of-interest

^†^Non-normal distribution, and median values are presented, and non-parametric Spearman’s rank correlation coefficient was used for comparison

### Intra-measurement repeatability

The mean or median CVs of repeatedly measured T1 values of each ROI in 28 subjects were 0.45–1.31% in MRF3k and 0.40–1.58% in MRF1.5k. The CV of T1 values was significantly higher in MRF1.5k than MRF3k in the right MCP, but the difference was only 0.65%. The mean or median CVs of T2 values were 0.55–2.50% and 0.90–3.13% for MRF3k and MRF1.5k, respectively. The CVs of T2 values were significantly higher in the left putamen, left thalamus and right thalamus in MRF1.5k than in MRF3k, but the differences were only 0.69, 0.98 and 0.85%, respectively (Table 4). Differences in CVs measured as an index of repeatability were found to be less than 1% in each of the ROIs.

**Table 4:** Mean and median coefficient of variation (CVs) for intra-measurement repeatability of MRF3k and MRF1.5k and the 95% confidence intervals (CI) as well as statistical difference (*p* values) for T1 (a) and T2 (b) values

ROIs	MRF3k		MRF1.5k		*p* value
	CVs of T1	95% CI	CVs of T1	95% CI	
(a)					
Left MCP	0.84	0.58–1.10	1.04	0.74–1.33	0.29
Right MCP	0.8	0.60–0.99	1.45	1.04–1.86	0.004*
Pons	1.31	0.89–1.72	0.83	0.59–1.07	0.06
Left caudate	0.95	0.63–1.27	0.91	0.58–1.23	0.85
Right caudate	0.95	0.65–1.24	0.94	0.70–1.18	0.97
Left putamen	0.59	0.42–0.77	0.76	0.54–0.98	0.26
Right putamen	0.51	0.37–0.64	0.66	0.47–0.86	0.17
Left pallidus	1.11	0.76–1.46	1.58	1.21–1.94	0.09
Right pallidus	0.94	0.60–1.27	1.15	0.85–1.46	0.34
Left thalamus	0.66	0.44–0.88	0.63	0.45–0.82	0.83
Right thalamus	0.45^†^	0.30–0.70^†^	0.40^†^	0.30–0.80^†^	0.48^†^
Corpus callosum	1.1	0.72–1.48	1.13	0.76–1.50	0.91
Left semiovale	0.50^†^	0.34–0.70^†^	0.75^†^	0.54–1.09^†^	0.07^†^
Right semiovale	0.66	0.44–0.88	0.86	0.56–1.16	0.07

ROIs	MRF3k		MRF1.5k		*p* value
	CVs of T2	95% CI	CVs of T2	95% CI	
(b)					
Left MCP	1.63	1.06–2.20	2.09	1.63–2.55	0.27
Right MCP	1.48	1.06–1.89	1.47	0.98–1.96	0.98
Pons	2.50	1.85–3.15	3.13	2.31–3.95	0.25
Left caudate	1.14	0.65–1.62	1.74	1.16–2.32	0.08
Right caudate	1.71	1.13–2.28	2.26	1.48–3.05	0.21
Left putamen	0.79	0.39–1.19	1.48	1.00–1.95	0.02*
Right putamen	1.06	0.68–1.44	1.08	0.70–1.45	0.95
Left pallidus	1.84	1.07–2.62	2.18	1.52–2.83	0.44
Right pallidus	1.36	0.95–1.78	1.78	1.24–2.31	0.29
Left thalamus	0.99	0.65–1.33	1.97	1.38–2.56	0.01*
Right thalamus	0.55^†^	0.30–1.26^†^	1.40^†^	0.94–2.10^†^	0.001^†^*
Corpus callosum	1.64	1.07–2.20	2.10	1.49–2.72	0.22
Left semiovale	0.65^†^	0.40–1.10^†^	1.00^†^	0.74–1.60^†^	0.33^†^
Right semiovale	0.70^†^	0.44–0.86^†^	0.90^†^	0.74–1.40^†^	0.24^†^

*MCP *middle cerebellar peduncle, *ROIs *regions-of-interest

^*^Asterisks indicate statistical significance

^†^Obelisks indicate non-normal distribution, and median values are presented, and non-parametric Wilcoxon test was used for comparison

## Discussion

In this study, MRF measurements with different numbers of echoes, and consequently scan lengths, were compared to examine inter-measurement differences and correlations of absolute T1 and T2 values using a relatively large number of subjects. The mean T1 and T2 values in this study were similar to those in a multi-center MRF study [10]. The values were strongly to very strongly correlated between MRF3k and MRF1.5k. However, the estimated T1 and T2 values were higher by 0.9% and 6.6%, respectively, in MRF1.5k than in MRF3k. Considering these biases, either one type of MRF should be consistently used to take advantage of the high repeatability of MRF scans presented in this study.

CVs, an index of intra-measurement repeatability, were less than 1.58% for T1 and 3.13% for T2. The CVs were significantly higher at the right MCP for T1 and at the left putamen and bilateral thalamus for T2 in MRF1.5k than in MRF3k, but the differences in CVs were less than 1% (see Table 4). These results confirmed that cutting the scan time in half has little effect on the repeatability. However, it should be noted that the acquisition parameters of MRF1.5k were not exactly the same as the first half of MRF3k. The same TR values were used, but the FAs were higher in MRF1.5k than in MRF3k (see Fig. 1). In addition, MRF1.5k had a slightly higher in-plane resolution that is considered advantageous, especially for small structures such as the substantia nigra, red nucleus, subthalamic nucleus, and habenula. However, this potential advantage was not exploited in the comparison with MRF3k because MRF1.5k images were down-sampled to match the resolution of MRF3k. The original pixel size of the MRF1.5k images was approximately 27% smaller (1–1.0^2^/1.17^2^), resulting in a lower signal-to-noise ratio (SNR), and ROIs were placed on the MRF3k T1 maps. The coregistration of MRF1.5k images to MRF3k images was a linear transform with in-plane translation and rotation. Resampling was conducted using bilinear interpolation, which will introduce a certain averaging effect, i.e., possible increase in SNR, but the average values of the ROI are the same except for the pixels at the edge of ROIs. Therefore, the effect of resampling on SNR is considered small. These procedures possibly caused higher CVs in MRF1.5k than in MRF3k.

Recently, a multi-center study that assessed the repeatability and reproducibility of 2D MRF using FISP, which is the same as MRF3k but used a 20-channel head coil, was reported [10]. Intra-scanner repeatability half-widths of the confidence intervals for relative deviations, which are calculated as 1.96 times the CVs, were in the range of 2.0–3.1% for T1 and 3.1–7.9% for T2 in solid tissue compartments. The CVs of MRF3k were 0.45–1.31% and 0.55–2.50% for T1 and T2 values, respectively, and our results concerning intra-scanner repeatability half-widths were 0.88–2.56% and 1.08–3.61%, showing similar high repeatability. The CVs of T2 were higher than those of T1, apparently due to the relatively narrow ranges of TR and TE, which reduce T2-related signal changes. Such limitations of this type of 2D-MRF measurement caused T2 to have lower repeatability than T1.

In a prior study, T2 measurements had greater variation than T1 measurements [9]. This difference was attributed to B1 variation that affects the measured T2 values more than T1 values in a previous MRF study [16]. Diffusion weighting caused by spoiler gradients may also lead to inaccurate T2 measurements in MRF [17]. However, both B1 and spoiler gradients were the same for MRF3k and MRF1.5k. These factors also explain the larger CVs in T2 than T1 but not for bias. Although it cannot be accounted for, no apparent trend was observed dependent on the averaged T2 values, and the bias was stable. These results support the consistent use of either MRF3k or MRF1.5k.

The proposed MRF1.5k scan reduced the scan time by half while retaining high repeatability comparable to that of the long scan, i.e., MRF3k [10]. This large reduction in scan time is highly advantageous in clinical practice. Quantitative imaging biomarkers are increasingly employed to investigate pathogenic processes and monitor therapeutic response [18], and MRF is expected to play an important role. Further reduction in scan time by a simultaneous multi-slice [19] and 3D [20] MRF as well as advanced reconstruction methods [21–23] will facilitate the use of MRF.

Our study has some limitations. First, no relaxometry method other than MRF was used to compare the absolute values. Second, the alignment of each image was performed in 2D mode so that slight subject motion could be corrected and the same ROIs could be applied for objective analysis; if a subject’s brain was shifted in up/down or rotated in the pitch or roll direction during imaging, such displacement could not be corrected, but no apparent misregistration was noticed by the raters. Third, we compared 4 scan conditions—echo numbers of 3000 vs. 1500 and in-plane resolution of 1.17 mm × 1.17 mm vs. 1 mm × 1 mm—in the phantom (see supplementary material) but not in the human subjects. This was because the 4 scan conditions were comparable in the phantom, and we intended to focus on MRF3k (1.17 mm × 1.17 mm) and MRF1.5k (1 mm × 1 mm) to reduce the total scan time and compare them in a relatively large number of human subjects. Fourth, ROIs may introduce rater biases, but ICCs between raters were good to excellent (0.77–0.98), and such biases are considered limited.

In conclusion, MRF3k and MRF1.5k had high repeatability and were strongly correlated this healthy-volunteer study; however, some measurement bias suggests that consistent use of one condition or the other would be optimal.

## Electronic supplementary material

Below is the link to the electronic supplementary material.

Supplementary file1 (DOCX 28 kb)

## Abbreviations

CI: Confidence interval
CV: Coefficient of variation
FA: Flip angle
ICC: Intraclass correlation coefficient
MCP: Middle cerebellar peduncle
MRF: Magnetic resonance fingerprinting
ROI: Region of interest
TE: Echo time
TR: Repetition time
