# Supplementary material for: Acceleration of 2D-MR fingerprinting by reducing the number of echoes with increased in-plane resolution: a volunteer study
Source: MAGMA. 2020 Apr 4;33(6):783–91. doi: 10.1007/s10334-020-00842-8 (PMC7669790; doi:10.1007/s10334-020-00842-8)
Supplement: Supplementary file 1 — Supplementary file1 (DOCX 28 kb) [file 10334_2020_842_MOESM1_ESM.docx]

Supplementary material. A phantom study of a comparison of MRF3k and MRF1.5k.

The acquisition parameters of MRF1.5k were developed based on those of MRF3k. To improve measurement quality, especially that of T2 values, the pattern of FA values was empirically modified as is illustrated in Figure 1 to compensate for the lower scan time. To check difference between 2 different FA patterns (original and modified), scans were conducted using a NIST/ISMRM system phantom that has known reference T1 and T2 values [S1] using original MRF3k, original MRF1.5k and modified MRF1.5k scans in 1.0 mm resolution.

ROIs were placed to measure mean values (Table S1), and they were compared using a non-parametric pair-wise Friedman test. Significant differences (*P* < 0.05) were found in the T2 values measured by original MRF3k (mean rank: 2.11) and original MRF1.5k (1.11) compared with the reference T2 values (3.22) and those measured using modified MRF1.5k (3.56) after a *post-hoc* test, whereas no significant difference was detected between the reference T2 values and those measured by modified MRF1.5k. Also, the original MRF1.5k was significantly worse than the original MRF3k. The absolute differences from the reference values were 0.21 – 9.39 %, 1.49 – 17.81 % and 1.23 – 10.31 % for modified MRF1.5k, regular MRF1.5k and regular MRF3k, respectively. Original MRF3k was found to be nearer to the reference values than original MRF1.5k, meaning that reduced number of echoes reduced accuracy of T2 measurements. However, the modified FA pattern was considered to compensate this reduction in accuracy of T2 measurements.

In T1 values, significant differences (*P* < 0.05) were found in all 6 combinations of pair-wise comparison by a *post-hoc* test. The mean ranks were near to the reference T1 value (4.00) in the order of original MRF1.5k (2.80), modified MRF1.5k (2.20) and original MRF3k (1.00), but the absolute differences from the reference values were 0.01 – 3.87 %, 1.52 – 5.81 % and 2.67 – 6.16 %, respectively. Although the differences were statistically significant, but they were relatively small compared with those of T2 values. Therefore, the modified FA pattern was adopted.

Because higher resolution is also important to measure small structures, scans with resolutions of both 1.17 mm and 1.0 mm were conducted for 5 times each using the same phantom using the original MRF3k and modified MRF1.5k. ROIs were placed on the arrays, and mean, standard deviation (SD) and coefficient of variation (CV) were measured.

The results are summarized in Table S2. The CV values are mostly less than 1 % with some higher values up to 1.6%. The correlation coefficients to the true values were higher than 0.99 in all scan conditions (Table S3). Based on these results, scans using MRF3k with in-plane resolution of 1.17 mm that has been used in former studies (8,10 in the main text) and MRF1.5k with in-plane resolution of 1.0 mm were compared in this study.

Table S1. Measured T1 and T2 values (ms) of the NIST phantom in 1.0 mm in-plane resolution.

|  | MRF3k  Original FA | MRF1.5k Original FA | MRF1.5k Modified FA | Reference values |
| --- | --- | --- | --- | --- |
| T1-1 | 1935.88 | 1988.75 | 1958.75 | 1989.00 |
| T1-2 | 1364.50 | 1403.13 | 1369.50 | 1454.00 |
| T1-3 | 925.75 | 946.56 | 946.00 | 984.10 |
| T1-4 | 667.75 | 682.19 | 679.25 | 706.00 |
| T1-5 | 466.50 | 477.50 | 487.00 | 496.70 |
|  |  |  |  |  |
| T2-1 | 541.25 | 520.63 | 542.50 | 581.30 |
| T2-2 | 361.88 | 331.63 | 365.63 | 403.50 |
| T2-3 | 261.50 | 252.75 | 264.00 | 278.10 |
| T2-4 | 186.75 | 180.00 | 193.75 | 190.94 |
| T2-5 | 141.00 | 135.25 | 145.63 | 133.27 |
| T2-6 | 94.16 | 91.13 | 97.25 | 96.89 |
| T2-7 | 62.45 | 61.25 | 67.40 | 64.07 |
| T2-8 | 45.85 | 45.19 | 48.00 | 46.42 |
| T2-9 | 30.20 | 29.81 | 31.90 | 31.97 |

Table S2. Measured T1 and T2 values (ms) in different resolutions and their stability evaluated by CV.

| ROIs | MRF3k  1.17 mm | | | MRF3k  1.0 mm | | | MRF1.5k  1.17 mm | | | MRF1.5k  1.0 mm | | | Reference values |
| --- | --- | --- | --- | --- | --- | --- | --- | --- | --- | --- | --- | --- | --- |
|  | Mean | SD | CV | Mean | SD | CV | Mean | SD | CV | Mean | SD | CV |  |
| T1-1 | 1897.27 | 5.11 | 0.3% | 1897.71 | 3.60 | 0.2% | 1920.73 | 1.94 | 0.1% | 1914.51 | 2.86 | 0.1% | 1989 |
| T1-2 | 1371.38 | 2.60 | 0.2% | 1366.33 | 2.17 | 0.2% | 1372.00 | 1.87 | 0.1% | 1371.16 | 1.02 | 0.1% | 1454 |
| T1-3 | 926.35 | 0.63 | 0.1% | 909.67 | 1.05 | 0.1% | 937.57 | 0.67 | 0.1% | 916.46 | 1.64 | 0.2% | 984.1 |
| T1-4 | 664.85 | 1.39 | 0.2% | 657.08 | 0.66 | 0.1% | 666.23 | 0.95 | 0.1% | 659.42 | 1.20 | 0.2% | 706 |
| T1-5 | 470.50 | 1.13 | 0.2% | 469.62 | 0.79 | 0.2% | 474.69 | 1.03 | 0.2% | 469.62 | 0.70 | 0.1% | 496.7 |
|  |  |  |  |  |  |  |  |  |  |  |  |  |  |
| T2-1 | 512.17 | 2.67 | 0.5% | 522.34 | 1.61 | 0.3% | 494.83 | 4.13 | 0.8% | 502.19 | 6.16 | 1.2% | 581.3 |
| T2-2 | 392.25 | 2.11 | 0.5% | 381.35 | 2.70 | 0.7% | 369.00 | 5.75 | 1.6% | 375.21 | 4.23 | 1.1% | 403.5 |
| T2-3 | 273.78 | 1.44 | 0.5% | 269.00 | 1.22 | 0.5% | 269.00 | 2.67 | 1.0% | 271.00 | 2.41 | 0.9% | 278.1 |
| T2-4 | 199.23 | 0.60 | 0.3% | 193.84 | 0.58 | 0.3% | 195.46 | 1.14 | 0.6% | 192.98 | 1.14 | 0.6% | 190.94 |
| T2-5 | 146.64 | 0.69 | 0.5% | 140.02 | 0.42 | 0.3% | 143.51 | 0.41 | 0.3% | 142.50 | 0.70 | 0.5% | 133.27 |
| T2-6 | 101.50 | 0.51 | 0.5% | 100.81 | 0.16 | 0.2% | 101.19 | 0.83 | 0.8% | 101.15 | 0.62 | 0.6% | 96.89 |
| T2-7 | 76.22 | 0.48 | 0.6% | 72.78 | 0.44 | 0.6% | 74.98 | 0.74 | 1.0% | 73.61 | 0.78 | 1.1% | 64.07 |
| T2-8 | 49.07 | 0.37 | 0.8% | 47.40 | 0.39 | 0.8% | 47.41 | 0.48 | 1.0% | 47.34 | 0.47 | 1.0% | 46.42 |
| T2-9 | 31.48 | 0.33 | 1.0% | 31.55 | 0.37 | 1.2% | 29.98 | 0.33 | 1.1% | 31.30 | 0.46 | 1.5% | 31.97 |

NB: MRF3k and MRF1.5k indicates scans used the original and modified FA patterns, respectively, as are described in the main text.

Table S3. Correlation coefficients to the reference T1 and T2 values of the NIST phantom.

|  | MRF3k 1.17 mm | MRF3k 1.0 mm | MRF1.5k 1.17 mm | MRF1.5k 1.0 mm |
| --- | --- | --- | --- | --- |
| T1 | 0.9999 | 0.9998 | 0.9998 | 0.9998 |
| T2 | 0.9968 | 0.9988 | 0.9967 | 0.9973 |

NB: MRF3k and MRF1.5k indicates scans used the original and modified FA patterns, respectively, as are described in the main text.

Reference

S1. Multi-Site, Multi-Vendor Comparison of T1 Measurement Using ISMRM/NIST System Phantom

Keenan K, Stupic K, Boss K, et al. ISMRM2016 abstract: 3290.
